# Interactions of Sulfate with Other Nutrients As Revealed by H_2_S Fumigation of Chinese Cabbage

**DOI:** 10.3389/fpls.2016.00541

**Published:** 2016-04-27

**Authors:** Martin Reich, Muhammad Shahbaz, Dharmendra H. Prajapati, Saroj Parmar, Malcolm J. Hawkesford, Luit J. De Kok

**Affiliations:** ^1^Laboratory of Plant Physiology, Groningen Institute for Evolutionary Life Sciences, University of GroningenGroningen, Netherlands; ^2^Department of Chemistry and Biochemistry, Worcester Polytechnic InstituteWorcester, MA, USA; ^3^Plant Biology and Crop Science Department, Rothamsted ResearchHarpenden, UK

**Keywords:** Brassica, hydrogen sulfide, sulfur deficiency, yield quality, mineral composition

## Abstract

Sulfur deficiency in plants has severe impacts on both growth and nutrient composition. Fumigation with sub-lethal concentrations of H_2_S facilitates the supply of reduced sulfur via the leaves while sulfate is depleted from the roots. This restores growth while sulfate levels in the plant tissue remain low. In the present study this system was used to reveal interactions of sulfur with other nutrients in the plant and to ascertain whether these changes are due to the absence or presence of sulfate or rather to changes in growth and organic sulfur. There was a complex reaction of the mineral composition to sulfur deficiency, however, the changes in content of many nutrients were prevented by H_2_S fumigation. Under sulfur deficiency these nutrients accumulated on a fresh weight basis but were diluted on a dry weight basis, presumably due to a higher dry matter content. The pattern differed, however, between leaves and roots which led to changes in shoot to root partitioning. Only the potassium, molybdenum and zinc contents were strongly linked to the sulfate supply. Potassium was the only nutrient amongst those measured which showed a positive correlation with sulfur content in shoots, highlighting a role as a counter cation for sulfate during xylem loading and vacuolar storage in leaves. This was supported by an accumulation of potassium in roots of the sulfur-deprived plants. Molybdenum and zinc increased substantially under sulfur deficiency, which was only partly prevented by H_2_S fumigation. While the causes of increased molybdenum under sulfur deficiency have been previously studied, the relation between sulfate and zinc uptake needs further clarification.

## Introduction

Understanding interactions between plant nutrients is essential for optimizing fertilization strategies and improving nutrient use efficiency in crops (Baxter, 2009; Maathuis, 2009; Reich et al., 2014a). Sulfur was recognized as an essential nutrient for crops more than a century ago (Bogdanov, 1899; Hart and Peterson, 1911) and since then it had shown to be involved in many vital processes in plants (Thompson, 1967; Hell, 1997; De Kok et al., 2005; Hawkesford and De Kok, 2006; Takahashi et al., 2011). In contrast to the intensive study of the uptake and assimilation of sulfur (reviewed e.g., by Leustek et al., 2000; Kopriva, 2006; Hawkesford, 2012; Honsel et al., 2012) and their interconnection with carbon and nitrogen metabolism (Kopriva et al., 2002) the interaction with other nutrients is less well-known.

As sulfur deficiency is becoming a constraint to yield in many cropping systems throughout the world (Zhao et al., 1999) it is momentous to uncover the effects on other elements, which determine the nutritional quality of crops. Numerous members of the Brassicaceae family are used as food and oil crops worldwide and Chinese cabbage has an increasing importance in many developing countries (Kawashima and Soares, 2003; Rakow, 2004; Park et al., 2005) and is highly nutritious (Moreno et al., 2002; Kawashima and Soares, 2003; Di Noia, 2014).

Interactions between nutrients may appear at different physiological levels. The uptake of nutrients from the soil solution by the roots represents the first level of possible interaction. Mineral nutrients are usually taken up in the form of soluble salts, i.e., as cations or anions. Differences in charge leads to antagonisms and synergisms between ions and many nutrient interactions may be driven by a balance of charge. An increased uptake of an anion may lead to the decrease of nutrients taken up as cations or an increase of another anion and *vice versa*. Additionally, ion transporters usually do not exclusively transport one single nutrient as their substrate but also translocate others with similar molecular structure, though usually with a lower affinity. Therefore the deficiency or complete absence of the preferred ion might lead to the transport and subsequent accumulation of another ion that would usually be outcompeted as a substrate. This is true, for example for sulfate transporters in the plasma membrane of roots, which have been shown to also transport selenate and molybdate (Shibagaki et al., 2002; Shinmachi et al., 2010). Due to its similar size, selenium may replace sulfur in many molecules (White et al., 2004).

Studies on nutrient interactions almost exclusively apply alterations of the rhizospheric concentration of the nutrient of interest to study the impact on uptake and metabolism of other nutrients. This always bears the risk of observing indirect effects due to changes in growth. Studies on sulfur offer the possibility of supplying plants with sulfur gases as an additional or sole source of reduced sulfur. In the present study H_2_S was used in concentrations that were below toxic levels but high enough to cover the bulk requirement of the plant for organic sulfur (also see Maas et al., 1987; Westerman et al., 2000). If sulfur is present in sufficient concentrations in the root medium, H_2_S fumigation typically leads to a partial down-regulation of sulfate uptake by the roots. However, if sulfur is absent in the root medium, H_2_S can serve as a source for sulfur and enable normal growth. In many industrial regions in the world significant amounts of H_2_S are present in the atmosphere and might have an impact on the nutritional quality of crops.

The aim of the present study was to examine the separate and interactive effects of rhizospheric and atmospheric sulfur nutrition on the tissue content and shoot-to-root partitioning of other essential macro- and micronutrients. The results will help to distinguish between nutrients that are directly affected by the presence or absence of sulfate and nutrients, which are coupled to the changes in growth and organic sulfur caused by different sulfur supply.

## Materials and methods

### Plant material, growth conditions, and growth analysis

*Brassica pekinensis* (Lour.) Rupr. cv. Kasumi F1 (Nickerson-Zwaan, Made, The Netherlands) was germinated in vermiculite. Ten day-old seedlings were grown in a 25% Hoagland nutrient solution (pH 5.9), consisting of 1.25 mM Ca(NO)_3_.4H_2_O, 1.25 mM KNO_3_, 0.25 mM KH_2_PO_4_, 11.6 μM H_3_BO_3_, 2.4 μM MnCl_2_.4H_2_O, 0.24 μM ZnSO_4_.7H_2_O, 0.08 μM CuSO_4_.5H_2_O, 0.13 μM Na_2_MoO_4_.2H_2_O, and 22.5 μM Fe^3+^-EDTA containing either 0.5 mM (+S) or 0 mM (-S) MgSO_4_.7H_2_O. Plants were grown in 13 l containers (10 sets of plants per container, three plants per set) in climate-controlled fumigation cabinets for 11 days and fumigated with 0 or 0.2 μl l^−1^ H_2_S. Day/night temperatures were 21/18°C, relative humidity was 60–70% and the photoperiod was 14 h at a photon fluence rate of 300 ± 20 μmol m^−2^ s^−1^ (within the 400–700 nm range) at plant height, supplied by Philips HPI-T (400 W) lamps.

For determination of the dry matter content fresh plant tissue was dried at 80°C for 24 h and stored in a desiccator for further use.

### Analysis of mineral nutrient content

Dried plant tissue (0.2–0.5 g) was digested with 5 ml of nitric acid/perchloric acid (87:13, v/v; 70% concentration, trace analysis grade; Fisher Scientific; Zhao et al., 1994). The digest solution samples were analyzed for mineral nutrients by inductively coupled plasma mass spectrometry (ICP-MS) and inductively coupled plasma atomic emission spectrometry (ICP-AES) analysis. Repeat samples were carried out every 10 samples; blanks and standard reference material (NIST 1567, a wheat flour) were used for quality control.

Inductively coupled plasma analysis was carried out using a 7500ce Octopole Reaction System ICP-MS apparatus (Agilent Technologies). The sample introduction system consisted of a micromist glass concentric nebulizer, quartz Scott-type double-pass spray chamber at 2°C, and nickel sample (1 mm) and skimmer (0.4 mm cones). Operating parameters were optimized daily using a tune solution containing 1 μg l^−1^ cerium, lithium, tellurium, and yttrium. Other instrument conditions were radiofrequency forward power of 1550, sample depth of 8.0 mm, carrier gas flow rate of 0.89 l min^−1^, reaction gas flow rate (H_2_) of 4 ml min^−1^ or (helium) of 4.5 ml min^−1^. An internal standard (500 μg l^−1^ germanium) was used to correct for signal drift. The analytical procedures gave satisfactory values for the standard reference materials.

Mineral nutrient contents were measured from dried material. These contents were multiplied with the average dry matter content to calculate the contents based on fresh weight.

### Statistical analysis

One-way-analysis of variance (ANOVA) was used to test for significant differences in growth parameters (Table 1) and an Unpaired Student's *t*-test to compare nutrient contents of the treatments (-S, H_2_S, -S H_2_S) with the control conditions (+S; **Table 3**). A two-way-ANOVA was performed to analyze the contribution of rhizosperic and atmospheric sulfur supply to the total variance in nutrient contents (**Table 4**). The changes in sulfur and potassium content were correlated using a linear regression (**Figure 2**). All analyses were performed using GraphPad Prism (GraphPad Software, San Diego, CA, USA).

**Table 1:** **The effect of sulfur deprivation (−S) and H_2_S fumigation on total biomass (fresh weight), shoot-to-root ratio and dry matter content (DMC) of shoot and roots of seedlings of Chinese cabbage**.

	**Total biomass (FW)**	**Shoot-to-root ratio**	**DMC shoot**	**DMC roots**
Control	3.14 ± 0.28a	7.64 ± 0.75a	6.63 ± 0.11a	6.72 ± 0.10a
−S	0.58 ± 0.31b	4.25 ± 0.41b	11.93 ± 0.30b	8.11 ± 0.59b
H_2_S	3.15 ± 0.34a	7.31 ± 1.04a	6.75 ± 0.05a	6.25 ± 0.12a
−S H_2_S	2.71 ± 0.42a	5.42 ± 0.99c	6.96 ± 0.86a	6.45 ± 0.14a

*Data represent the mean (± SD) of nine measurements with three plants in each. Data derived from Shahbaz et al. (2014). Values with different letters are significantly different(p < 0.05; one-way-ANOVA, Tukey's multiple comparison as post-hoc test)*.

## Results and discussion

Understanding all interactions between plant nutrients remains a challenge (Baxter, 2009; Maathuis, 2009) but is essential to improve nutrient use efficiency of agricultural and horticultural systems (Reich et al., 2014a). A major constraint in studying nutrient-nutrient interactions is the effect of nutrient availability on plant growth. Decreasing the tissue content of an essential nutrient below a critical level will lead to growth impairment and consequently the changes in content of other nutrients can be a direct cause of the absence of this nutrient or an indirect result of the impaired growth. The regulation of sulfate uptake and sulfur metabolism is presumed to be interconnected with plant development (Hawkesford, 2012).

**Figure 1:**
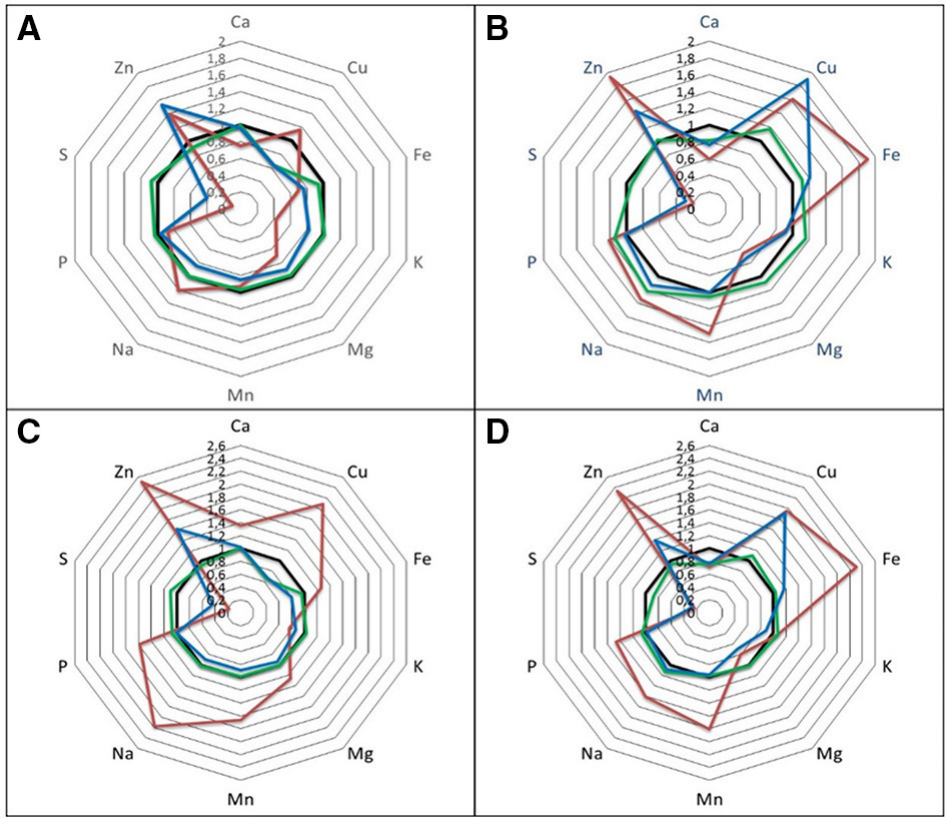
**The effect of sulfur deprivation and H_**2**_S fumigation on mineral nutrient content in shoot and roots of seedlings of Chinese cabbage**. Radar diagrams showing response ratios relative to control conditions. Shoot **(A,C)**; roots **(B,D)**; dry weight basis **(A,B)**; fresh weight basis **(C,D)**. Control (black); H_2_S (green); −S (red); −S + H_2_S (blue). Molybdenum was excluded from this figure due to its extraordinary large changes. For absolute contents see Table 2.

This study presents results obtained from an experimental set-up in which inorganic sulfur status was uncoupled from effects on growth and the organic sulfur pool. H_2_S fumigation serves as a reduced sulfur source to plants and leads to a replenishing of the organic sulfur fraction in shoots and, to a lesser extent, also in the roots whilst leaving inorganic sulfur pools (*viz*. sulfate) largely unaffected (Shahbaz et al., 2014). This creates a situation in which effects of sulfate status can be disentangled from effects of growth (Table 1; De Kok et al., 2007). In sulfur deficiency, the increase of calcium, copper, iron, magnesium, manganese, sodium, and phosphorus in shoots on a fresh weight basis was completely reversed if plants were supplied with H_2_S (Figure 1, Tables 2, 3). On a dry weight basis all these nutrients, except copper and sodium, were actually decreased. A two-way ANOVA showed that the variation in zinc and molybdenum content was mainly caused by rhizospheric sulfur supply (Table 4). The large increase of molybdenum is known to be caused by the affinity of sulfate transporters for molybdate (Leggett and Epstein, 1956; Fitzpatrick et al., 2008; Shinmachi et al., 2010). The other way around, an excessive sulfur fertilization can lead to molybdenum deficiency (MacLeod et al., 1997). Interestingly, H_2_S exposure counteracted the increase of molybdenum under sulfur deficiency, although it did not completely reverse it (Figure 1, Tables 2, 3). It is a well-known phenomenon that atmospheric, sub-lethal H_2_S concentrations enable plants to maintain sufficient levels of organic sulfur compounds in leaves but do not necessarily lead to a complete down-regulation of gene expression of the sulfate transporters and sulfate uptake capacity (De Kok et al., 1997; Buchner et al., 2004; Koralewska et al., 2008). The effect of H_2_S on molybdenum levels in the present study could be due to this partial down-regulation of the sulfate transporters or to an effect of growth as proposed for the other nutrients. The strong effect of sulfur deficiency observed on zinc found in the present study is a less studied phenomenon. One possible explanation for this observation is a change in rhizosphere pH. It is well-known that zinc uptake negatively correlates with rhizosphere pH (Lucas and Davis, 1961; Marschner, 1993), which is the likely reason for higher zinc uptake under ammonium nutrition and phosphorus deficiency which both lead to an acidification of the rhizosphere (Alloway, 2009; Reich et al., 2016). Measurements with H^+^-electrodes showed that sulfur deficiency also leads to a lower pH at the roots of *B. pekinensis* seedlings (Reich et al., 2014b), which could increase zinc uptake. Another possibility for the increased content of transition metals under sulfur deficiency, and a prevention of such by H_2_S, is their reactivity with and mutual detoxification by reduced sulfur compounds. Particularly cysteine-rich polypeptides possessing sulfhydryl groups (-SH) are highly reactive with transition metals (Steffens, 1990). Under sulfur deficiency these compounds are less abundant while H_2_S fumigation usually leads to a restock or even higher concentrations (Buchner et al., 2004). This might explain why copper levels are recovered by H_2_S fumigation (Figure 1, Table 2) but not why zinc levels are still higher under sulfur deficiency and H_2_S fumigation. Both, molybdenum and zinc, are co-factors of important enzymes and zinc is involved in auxin biosynthesis (Mendel and Hänsch, 2002; Broadley et al., 2007). The metabolic consequences of an increase of these micronutrients under sulfur deficiency should be further investigated.

**Table 2:** **The effect of sulfur deprivation and H_2_S fumigation on mineral nutrient content in [μmol g dry weight^−1^] of shoot and roots of seedlings of Chinese cabbage**.

**Shoot**	**Control**	**−S**	**H_2_S**	**−S H_2_S**
Ca	799 ± 48	600 ± 16	792 ± 17	770 ± 31
Cu	0.18 ± 0.05	0.21 ± 0.02	0.12 ± 0.01	0.12 ± 0.01
Fe	1.61 ± 0.24	1.13 ± 0.07	1.52 ± 0.07	1.23 ± 0.21
K	2046 ± 137	870 ± 31	2070 ± 14	1683 ± 65
Mg	203 ± 14	142 ± 3	199 ± 7	182 ± 2
Mn	2.29 ± 0.06	2.12 ± 0.10	2.22 ± 0.15	1.94 ± 0.16
Mo	0.06 ± 0.004	0.40 ± 0.03	0.05 ± 0.002	0.21 ± 0.01
Na	9.11 ± 1.30	11.03 ± 0.38	9.10 ± 0.41	7.73 ± 0.36
P	221 ± 16	193 ± 5	232 ± 11	212 ± 1
S	279 ± 17	29 ± 2	301 ± 17	113 ± 9
Zn	0.33 ± 0.05	0.46 ± 0.02	0.30 ± 0.03	0.50 ± 0.02
**Roots**	+**S**	−**S**	+**S H**_2_**S**	−**S H**_2_**S**
Ca	487 ± 42	289 ± 15	397 ± 37	371 ± 21
Cu	0.38 ± 0.02	0.61 ± 0.01	0.44 ± 0.02	0.72 ± 0.11
Fe	17.8 ± 2.6	34.0 ± 1.6	19.9 ± 5.1	21.6 ± 2.0
K	1681 ± 5	1498 ± 59	1944 ± 164	1544 ± 44
Mg	186 ± 11	122 ± 4	202 ± 14	135 ± 2
Mn	19.7 ± 1.7	29.4 ± 1.3	20.8 ± 3.9	19.7 ± 1.6
Mo	0.07 ± 0.001	0.96 ± 0.07	0.08 ± 0.001	0.45 ± 0.02
Na	8.4 ± 0.4	11.3 ± 0.92	10.3 ± 1.1	9.5 ± 0.6
P	309 ± 4	375 ± 10	352 ± 35	316 ± 3
S	351 ± 14	68 ± 1	330 ± 29	100 ± 3
Zn	0.72 ± 0.09	1.40 ± 0.09	0.73 ± 0.07	1.04 ± 0.02

*Data represent the mean (± SD) of three measurements with three plants in each. Relative responses and significance are shown in Table 3*.

**Table 3:** **Relative effect of sulfur deficiency (–S), H_2_S fumigation on the content of mineral nutrients in shoot and roots of seedlings of Chinese cabbage**.

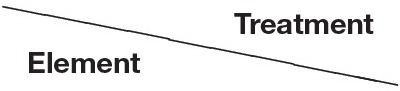	**Shoot (dry weight basis)**			**Roots (dry weight basis)**			**Shoot (fresh weight basis)**			**Roots (fresh weight basis)**		
	**−S**	**H_2_S**	**−S H_2_S**	**−S**	**H_2_S**	**−S H_2_S**	**−S**	**H_2_S**	**−S H_2_S**	**−S**	**H_2_S**	**−S H_2_S**
Ca	25^**^	1n.s.	1n.s.	41^**^	19^*^	24^*^	35^***^	1n.s.	2n.s.	28^**^	24^*^	27^**^
Cu	16n.s.	35n.s.	36n.s.	62^***^	18^**^	92^**^	109^**^	34n.s.	32n.s.	95^***^	10n.s.	84^**^
Fe	30^*^	6n.s.	24n.s.	91^***^	26n.s.	21n.s.	26n.s.	4n.s.	20n.s.	130^***^	4n.s.	16n.s.
K	57^***^	1n.s.	18^*^	11^**^	16^*^	8^**^	23^**^	3n.s.	14^*^	8^*^	8n.s.	12^**^
Mg	30^**^	2n.s.	10n.s.	34^***^	9n.s.	27^**^	26^**^	0	6n.s.	21^**^	1n.s.	30^***^
Mn	7n.s.	3n.s.	15^*^	50^**^	5n.s.	0	67^***^	1n.s.	11n.s.	81^***^	2n.s.	4n.s.
Mo	575^***^	19^*^	250^***^	1300^***^	23^***^	553^***^	1112^***^	17n.s.	267^***^	1589^***^	14n.s.	527^***^
Na	21n.s.	0	15n.s.	34^**^	22^*^	13n.s.	118^***^	2n.s.	11n.s.	62^**^	13n.s.	9n.s.
P	12^*^	4n.s.	4n.s.	22^***^	14n.s.	2n.s.	58^***^	7n.s.	1n.s.	47^***^	6n.s.	2n.s.
S	90^***^	8n.s.	59^***^	81^***^	6n.s.	72^***^	81^***^	10n.s.	57^***^	77^***^	12n.s.	73^***^
Zn	40^*^	9n.s.	53^**^	94^***^	1n.s.	45^**^	150^***^	7n.s.	61^**^	134^***^	6n.s.	39^**^

Data expressed as relative change in % to control levels. Relative increase compared to the control is accentuated in orange, relative decrease in blue. Significant difference from the control is indicated by bold font and coloration (Unpaired Student's t-test on original values;

* p < 0.05,

** p < 0.01,

*** *p < 0.001)*.

**Table 4:** Results of a two-way-ANOVA showing the contribution of rhizopsheric (R) and atmospheric (A) sulfur supply and their interaction (I) to the total variance in mineral nutrient content in shoot and roots of seedlings of Chinese cabbage (%; ^*^p < 0.05, ^**^p < 0.01, ^***^p < 0.001; Bonferroni's multiple comparison as ***post-hoc*** test).

	**Dry weight basis**						**Fresh weight basis**					
	**Shoot**			**Roots**			**Shoot**			**Roots**		
	***R***	***A***	***I***	***R***	***A***	***I***	***R***	***A***	***I***	***R***	***A***	***I***
Ca	42	23	27	56	0	33	36	28	31	38	20	29
*p*-value	^***^	^***^	^**^	^***^	n.s.	^**^	^***^	^***^	^***^	^**^	*	^**^
*F*-value	37.9	20.5	24.2	39.6	0.1	23.3	59.3	46.8	52.3	18.8	10.0	12.8
Cu	2	73	3	80	10	1	21	53	20	92	0	1
*p*-value	n.s.	^***^	n.s.	^***^	*	n.s.	^***^	^***^	^***^	^***^	n.s.	n.s.
*F-value*	0.9	26.2	0.9	64.7	7.9	0.5	31.5	78.7	29.2	109.1	0.0	1.7
Fe	65	0	4	43	14	28	2	45	31	41	25	28
*p-value*	^**^	n.s.	n.s.	^**^	*	^**^	n.s.	^**^	^**^	^***^	^***^	^***^
*F*-value	16.7	0.0	1.0	24.4	8.2	15.9	0.7	16.2	11.3	53.2	31.6	36.0
K	64	18	16	57	17	8	81	8	2	11	11	56
*p*-value	^***^	^***^	^***^	^***^	*	n.s.	^***^	*	n.s.	n.s.	n.s.	^**^
*F value*	304.0	87.1	77.4	31.4	8.9	4.4	75.9	7.7	2.2	3.9	3.9	20.4
Mg	61	13	19	91	4	0	16	39	39	86	2	4
*p*-value	^***^	^**^	^**^	^***^	*	n.s.	^**^	^***^	^***^	^***^	n.s.	n.s.
*F*-value	71.7	15.4	22.2	152.1	7.5	0.1	17.8	44.3	44.2	88.6	2.4	3.8
Mn	46	13	3	24	23	36	20	39	37	28	34	31
*p*-value	*	n.s.	n.s.	*	*	^**^	^***^	^***^	^***^	^***^	^***^	^***^
*F*-value	9.6	2.8	0.7	10.6	10.2	16.0	56.4	108.8	103.3	35.4	42.4	38.7
Mo	76	13	10	75	12	13	57	22	20	66	16	17
*p*-value	^***^	^***^	^***^	^***^	^***^	^***^	^***^	^***^	^***^	^***^	^***^	^***^
*F*-value	727.8	120.9	96.2	842.2	131.6	148.7	737.5	281.0	259.2	777.7	193.3	203.9
Na	1	40	39	19	0	53	25	36	38	32	15	43
*p*-value	n.s.	^**^	^**^	*	n.s.	^**^	^***^	^***^	^***^	^**^	^**^	^***^
*F*-value	0.4	15.4	15.2	5.4	0.0	15.2	110.9	162.0	170.6	25.3	12.3	34.3
P	52	20	3	6	2	69	28	26	43	23	27	45
*p*-value	^**^	*	n.s.	n.s.	n.s.	^**^	^***^	^***^	^***^	^***^	^***^	^***^
*F*-value	16.6	6.4	1.1	2.1	0.6	24.1	85.4	79.4	129.2	35.4	42.4	70.5
S	92	5	2	98	0	1	93	5	1	98	0	1
*p*-value	^***^	^***^	^**^	^***^	n.s.	*	^***^	^***^	*	^***^	n.s.	*
*F*-value	862.5	51.6	17.8	743.4	0.4	7.7	739.4	39.0	6.9	729.6	2.8	10.3
Zn	87	0	4	76	9	11	73	14	11	62	20	16
*p*-value	^***^	n.s.	n.s.	^***^	^**^	^**^	^***^	^***^	^***^	^***^	^***^	^***^
*F*-value	75.8	0.2	3.6	131.6	16.2	18.3	287.9	56.6	41.6	198.1	63.6	50.3

**Figure 2:**
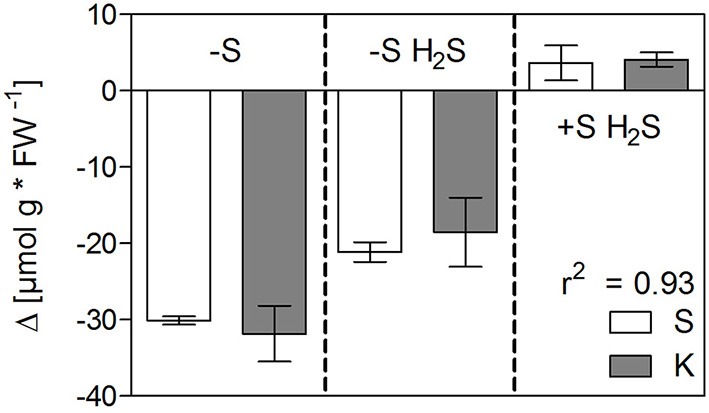
**Strong positive correlations between changes in sulfur and potassium contents in response to sulfur deficiency and H_**2**_S fumigation in the shoot of seedlings of Chinese cabbage**. The values for sulfur are multiplied by two to account for the divalency of sulfate. Data represent the mean (±SD) change in content relative to control conditions of three measurements with three plants in each. The goodness of fit (*r*^2^) of a linear regression is indicated.

**Figure 3:**
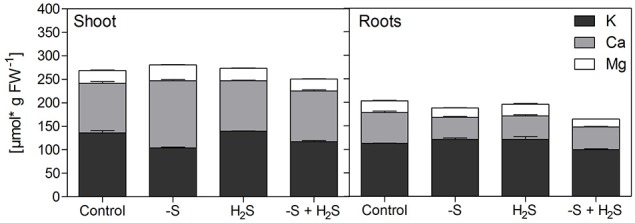
**The effect of sulfur deficiency and H_**2**_S fumigation on the cation balance in shoot and roots of seedlings of Chinese cabbage**. Data represent the mean (±SD) of the contents of potassium (K), calcium (Ca), and magnesium (Mg) in shoot (left) and root material (right) of three measurements with three plants in each. The contents of Ca and Mg were doubled to account for their divalency.

The only nutrient besides sulfur itself that significantly decreased in concentration in shoots under sulfur deficiency on both fresh and dry weight basis was potassium and this decrease was only partly reversed by H_2_S fumigation. Interestingly, potassium decreased to about the same extent as sulfur, if its content was multiplied by two in order to take the divalency of sulfate into account (Figure 2). Additionally, variation in potassium content in shoots was mainly caused by rhizospheric sulfur supply (Table 4). Potassium therefore seemed to compensate for the changes in sulfate and to play the role of a counter-ion. This is supported by studies on isolated vacuoles (Kaiser et al., 1989). Sulfate application also increased potassium levels in e.g., alfalfa (Razmjoo and Henderlong, 1997). In studies with Norway spruce potassium, magnesium, and manganese were increased in the endodermis and mesophyll cells if sulfate was increased due to SO_2_ fumigation. This led the authors to postulate that those cations act as counter-ions for sulfate accumulation in vacuoles (Slovik et al., 1996; Bäucker et al., 2003). While potassium was decreased together with sulfur in shoots under sulfur deficiency, calcium was increased (Figure 1, Table 3). Potassium, calcium, and magnesium are known to behave antagonistically in many cases due to their common positive charge, especially at the level of uptake (Jakobsen, 1993; Marschner, 2012). Indeed, contents of the single cations in the shoot differed proportionally more between sulfur sufficient and deprived conditions, than their sum (Figure 3).

The changes in growth due to the manipulation of rhizospheric and atmospheric sulfur supply seem to be the main driver of the content of most nutrients with the few above-mentioned exceptions which are linked to clear physiochemical mechanisms. However, looking at the changes in root tissue (Figure 1, Table 3) indicates a more complex picture. The relationship discussed above between sulfur and potassium was not found here. Instead, a relative accumulation of potassium was observed while calcium and magnesium decreased. However, the sum of decrease of the two cations was not enough to compensate for the decrease in sulfur, as it was for potassium in shoots. We assume that the xylem loading of potassium and its translocation to the shoot are partly determined by the amount of sulfate translocated and accumulating in the shoot, while potassium uptake by the roots is not. Therefore, potassium accumulates in the roots when sulfate is absent.

Some authors proposed a cross-talk between sulfur and iron uptake and metabolism (Forieri et al., 2013) due to the cooperative role of both nutrients in plant metabolism, for example in iron-sulfur clusters of proteins in the electron transport chain. In the present study, however, no significant changes in iron in leaves were observed (on a fresh weight basis) under sulfur deficiency, while sulfur decreased at 81%. On the contrary, in roots a 130% increase in iron was observed under sulfur deficiency, possibly indicating a declined sink strength of the shoot for iron due to the lack of sulfur. H_2_S completely reversed this effect. Also Zuchi et al. (2015) observed a decrease of iron in plants subjected to sulfur deficiency, however, here the content of the nutrients was expressed on a plant basis. As usual, sulfur deficiency led to severe impairment of growth in that study and a lower nutrient content on a plant level is to be expected. Dividing the iron content by the dry plant biomass given by Zuchi et al. (2015) revealed that that iron content was indeed also decreased by 55% on a dry weight basis, which was comparable to the decrease of 30% observed in the current study (Table 2). This decrease, however, disappeared if the large increase in dry matter content upon sulfur deficiency (Table 1) was taken into account and the content was calculated on a fresh weight basis, which presents a better estimation of the actual concentration (Table 2).

While increases in manganese, sodium, phosphorus, and zinc due to sulfur deficiency were prevented by H_2_S fumigation in shoot and roots, the increased copper levels in roots remained completely unaffected. Both copper and zinc increase the uptake of sulfate (Shahbaz et al., 2010; Stuiver et al., 2014) and, as the present study shows, sulfate deprivation in turn led to an increase in concentration of these transition metals in root and shoot tissues under non-toxic concentrations of these micronutrients in the growing medium. Exposure with H_2_S partly ameliorated this effect of sulfur deficiency, however, differently for copper and zinc. In response to H_2_S exposure, sulfur deficient plants showed copper levels in the shoot similar to that of sulfur sufficient plants. In the roots however, the increased copper levels were still maintained. The different interactions of zinc and copper with sulfate uptake and assimilation need further clarification.

## Conclusions

Sulfur deficiency has a diverse impact on the whole ionome of *B. pekinensis* with important implications for yield quality. By combining atmospheric and rhizospheric sulfur supply we were able to distinguish between nutrients on the basis of their direct or indirect interaction with the presence of sulfate. H_2_S fumigation with simultaneous sulfate deprivation revealed that most nutrients change due to growth impairment and changes in dry matter content under sulfur deficiency, rather than a direct interaction with sulfate. Potassium was the only nutrient that was decreased together with total sulfur under sulfur deficiency and showed a strong positive correlation with sulfur content. As sulfate represents the bulk part of total sulfur, these results suggests that potassium acts as the main counter-ion for the characteristically high sulfate levels in leaves of *Brassica*. Besides molybdenum also zinc, a crucial nutrient for human nutrition, was strongly increased by sulfur deficiency independent of changes in growth. Lower root surface pH under sulfur deficiency and a lower abundance of organic sulfur compounds, which could react with zinc are possible mechanisms.

## Author contributions

The experimental set up was designed by LD, MR, MS, and MH. The data for this study was acquired by MS and SP, analyzed by MR and interpreted by MR, LD, and MH. The manuscript was written by MR and LD with input from all other authors. DP practically supported the work. All authors gave their final approval for publication as well as agree to be accountable for the accuracy and integrity of the work.

### Conflict of interest statement

The authors declare that the research was conducted in the absence of any commercial or financial relationships that could be construed as a potential conflict of interest.
